# UTILIZATION OF USUAL SOURCE OF CARE AND HEALTH LITERACY AMONG OLDER ADULTS WITH HYPERTENSION

**DOI:** 10.1093/geroni/igae098.2466

**Published:** 2024-12-31

**Authors:** Dahye Hong, Jennifer Kim, Bada Kang

**Affiliations:** Yonsei University, Seoul, Republic of Korea; Yonsei University, Seoul, Republic of Korea; Yonsei University, Seoul, Republic of Korea

## Abstract

Health literacy (HL) is crucial for health outcomes in older adults with chronic conditions because it influences disease-related knowledge and medication adherence. Although the usual source of care (USC) is recognized as an influencing factor for HL, little is known about the association between the utilization of USC and health literacy among older adults with hypertension. This study examined the relationship between the utilization of USC and HL among adults aged 65 years or older with hypertension (N = 1,482 women and 1,064 men) using data from the Korea Health Panel Survey 2021. Three analyses were performed to examine the association between the utilization of USC and HL after adjusting for sociodemographic and health-related characteristics. The results of logistic regression, showed a negative association between the absence of USC and HL. When HL was divided into three levels (inadequate, problematic, and sufficient), the multinomial regression analysis indicated that the absence of USC demonstrated a more pronounced negative association with higher levels of HL. Linear regression analysis revealed a negative association between the absence of USC and all domains of health literacy (i.e., healthcare, disease prevention, and health promotion). Disease prevention, which is known to be associated with the prevention of multimorbidity, exhibited the most pronounced negative association with the absence of USC. These findings have implications for policymakers, as promoting the utilization of USC can be an effective approach for improving HL in older adults with hypertension and will be beneficial in preventing the incidence of multimorbidity among vulnerable populations.

